# HOME HEALTH CLINICIAN PERSPECTIVES ON ASSESSING AND SUPPORTING DEMENTIA CAREGIVERS

**DOI:** 10.1093/geroni/igae098.1232

**Published:** 2024-12-31

**Authors:** Julia Burgdorf, Margaret McDonald, Kathryn Bowles

**Affiliations:** VNS Health, New York, New York, United States; Visiting Nurse Service of New York, New York, New York, United States; VNS Health, New York, New York, United States

## Abstract

Home Health Care (HHC) delivers skilled services via visits to patients’ homes. One-third of HHC patients have dementia and caregivers play a critical role in their care. Yet, it is difficult to assess caregivers’ training/support needs during HHC. As part of ongoing formative research aimed at developing a dementia caregiver assessment intervention for HHC, we convened semi-structured group interviews with 16 frontline staff from a large, urban HHC agency—Registered Nurses (n=5), Physical Therapists (n=5), and Social Workers (n=6). Staff shared their perspectives on current communication with caregivers and reacted to a proposed health information technology (HIT) solution for assessing caregiver needs. Participants highlighted social work’s critical role in connecting caregivers with community resources to bridge the gap of “a short-term benefit trying to meet long-term needs”. Staff emphasized the importance of gathering information regarding dementia patients’ complex caregiving networks (encompassing family, paid attendants, and HHC aides with varying schedules and task loads) but noted there were few options for storing and sharing this information in the patient record. Respondents expressed enthusiasm for an HIT-driven assessment tool linked to the patient record and requested that such a tool (1) come with a repository of community resources, (2) support information sharing among the care team and between episodes, and (3) include education for caregivers on dementia symptoms and the scope of HHC. Findings shed light on current barriers and opportunities for supporting dementia caregivers in HHC and reveal practical considerations for designing an intervention that will integrate with existing HHC workflows.

